# Improved multi-objective artificial bee colony algorithm-based path planning for mobile robots

**DOI:** 10.3389/fnbot.2023.1196683

**Published:** 2023-06-01

**Authors:** Qiuyu Cui, Pengfei Liu, Hualong Du, He Wang, Xin Ma

**Affiliations:** School of Mechanical Engineering and Automation, University of Science and Technology Liaoning, Anshan, Liaoning, China

**Keywords:** mobile robots, path planning, multi-objective artificial bee colony algorithm, Bio-inspired algorithm, multi-objective optimization

## Abstract

Mobile robots are widely used in various fields, including cosmic exploration, logistics delivery, and emergency rescue and so on. Path planning of mobile robots is essential for completing their tasks. Therefore, Path planning algorithms capable of finding their best path are needed. To address this challenge, we thus develop improved multi-objective artificial bee colony algorithm (IMOABC), a Bio-inspired algorithm-based approach for path planning. The IMOABC algorithm is based on multi-objective artificial bee colony algorithm (MOABC) with four strategies, including external archive pruning strategy, non-dominated ranking strategy, crowding distance strategy, and search strategy. IMOABC is tested on six standard test functions. Results show that IMOABC algorithm outperforms the other algorithms in solving complex multi-objective optimization problems. We then apply the IMOABC algorithm to path planning in the simulation experiment of mobile robots. IMOABC algorithm consistently outperforms existing algorithms (the MOABC algorithm and the ABC algorithm). IMOABC algorithm should be broadly useful for path planning of mobile robots.

## 1. Introduction

As mobile robots are used in a wide range of applications in the home, defense, medical, and food service industries, they are being given more and more functions (Liu et al., 2022; Shi et al., 2023). Path planning is a key technique in mobile robots. Mobile robot path planning is to find an optimal collision-free path from the start point to the target point for a mobile robot in an environment with multiple obstacles according to a relevant criterion (Zhang et al., 2018). A good path-planning strategy ensures that mobile robots can complete their assigned tasks safely and efficiently.

Several path planning methods have been developed for application to mobile robot path planning, and these methods can be classified as potential field method, sampling method, and swarm intelligence method. The core of the potential field method is to consider the motion of a mobile robot in the environment as a kind of robot motion in a virtual artificial force field. Mohamed et al. (2011) improved the artificial potential field method to solve the problem of mobile robot path planning which is prone to fall into local optimum. Chen et al. (2015) improved the artificial potential field method by adding chaotic optimization methods to solve the local minima and target unreachability problems. Liu et al. (2021) proposed an adaptive path planning method with dual potential field fusion that can satisfy the path planning of mobile robots in the state space with different obstacles and different velocities. Lee et al. (2017) proposed NP-APF, which is a virtual target point in an obstacle-free environment to overcome the local minima and path inefficiency problems. Hou et al. (2017) proposed an improved artificial potential field (IAPF) method that solves the problems of the traditional APF method in robot path planning under different conditions and avoids the trap problem caused by local minima.

The core of the sampling method is to select a finite number of unstructured points in the configuration space and to establish connections between these points. The RRT algorithm is a sampling-based path planning algorithm that addresses the drawbacks of the RRT algorithm not applying to narrow spaces, dynamic environments, and slow convergence (LaValle and Kuffner, 2001). Kuffner (2000) proposed the RRT-connect algorithm, which introduced the greedy expansion idea on top of the bidirectional RRT algorithm to improve the convergence speed of the algorithm. Karaman and Frazzoli (2011) improved the RRT^*^ algorithm by adding asymptotically optimal properties to guarantee the quality of the paths while preserving the probabilistic completeness of the RRT algorithm. Nasir et al. (2013) proposed the RRT^*^-smart algorithm to achieve a sample-only search by heuristic sampling, which accelerates the convergence speed. Gammell et al. (2014) proposed the Informed-RRT^*^ algorithm to limit the node sampling range and improve the optimal path convergence speed by generating a subset of ellipsoidal samples.

Since the above methods have many shortcomings, researchers have started to study mobile robot path planning based on Bio-inspired algorithms, mainly including particle swarm algorithms, ant colony algorithms, genetic algorithms, and neural networks, and have achieved many results (Dorigo et al., 1996; Ghatee and Mohades, 2009; Mo and Xu, 2015; Liu et al., 2020). For example, Gunji et al. (2019) successfully obtained a mobile robot roadmap by a hybrid algorithm like cuckoo search and bats, and the algorithm was simulated and tested by a mobile robot in a real workspace. Hosseininejad and Dadkhah (2019) have designed a novel cuckoo search algorithm for the problem of dynamic path planning. Fakoor et al. (2016) proposed a fuzzy control algorithm based on the Markov decision process in which the obstacle avoidance requirement of a mobile robot was successfully implemented. Wang et al. (2016) proposed a genetic bee colony algorithm for the global path-planning problem of mobile robots. Nazarahari et al. (2019) have designed a novel genetic algorithm for path planning of multiple mobile robots in a complex environment map. This algorithm performs path planning in such a way that the path length can be reduced with enhanced safety. The principle is to add several crossover factors and multiple variation operators to the basic genetic algorithm. Qu et al. (2013) proposed a novel genetic algorithm, and the results showed that the convergence speed of the algorithm was enhanced and its search efficiency was improved. And the algorithm can also be well-applied to global path planning for multiple mobile robots. Wang et al. (2012) proposed a hybrid algorithm combining fuzzy logic and neural network, which can successfully bring the mobile robot to the task point under an unknown map. Lamini et al. (2018) devised an improved homo-neighborhood intersection operator that can generate feasible paths with better fitness values, successfully improved the problem of premature convergence of the algorithm, and was well-used in mobile robot path planning. Contreras-Cruz et al. (2015) fused evolutionary planning algorithms in artificial bee colony algorithms and thus obtained a novel algorithm that refines the feasible paths. To verify the authenticity of the algorithm, they conducted test experiments using a mobile robot in a real working environment, and the results confirmed the authenticity of the algorithm and good performance of the algorithm.

In summary, we can see that a very large number of intelligent algorithms have been applied to solve problems related to path planning for mobile robots, and all of them have achieved good results. However, the current research is basically for the single-objective path planning problem, while the mobile robot path planning is a constrained multi-objective optimization problem (Jeddisaravi et al., 2016). We have to consider how to study a more practical and effective way to solve this multi-objective problem.

In 2005, Turkish scholars Karaboga and Basturk first proposed the artificial bee colony algorithm (ABC), the basic idea of which was inspired by the honey harvesting task of bee colonies (Karaboga, 2005). The ABC algorithm has been favored by many scholars due to its advantages such as fast convergence and excellent algorithm performance in solving the problem. As the artificial bee colony algorithm was extended for solving multi-objective problems, the MOABC algorithm was then widely used in various fields. However, multi-objective artificial swarm algorithm-based path planning for mobile robots is rarely reported, so the contribution of this paper is as follows.

1. An improved MOABC algorithm is proposed.
2. The improved MOABC algorithm is applied to solve the mobile robot path planning to verify the effectiveness of the IMOABC algorithm.

The rest of this paper is organized as follows. Section 2 presents the relevant background knowledge. Section 3 presents the improvement and validation of the MOABC algorithm. Section 4 applies the improved MOABC algorithm to mobile robot path planning and validates it with experimental simulations. Section 5 summarizes the entire text.

## 2. Related background knowledge

### 2.1. MOABC algorithm

Since its introduction in 2005, the Artificial Bee Colony algorithm (ABC) has been favored by many scholars due to its unique advantages. The basic MOABC algorithm was first proposed by Hedayatzadeh et al. (2010). The MOABC algorithm consists of seven main actions: initializing the honey bee population, foraging for honey bees, generating new food sources, evaluating food source mechanisms, onlooker bees foraging, scouting for bees foraging, and local optimization for external archive. One of the main roles of the external archive is to keep and maintain records of the optimal Pareto solutions found up to now.

The following are the specific steps of the MOABC algorithm implementation:

1. Initialization: the initialization of each parameter and external archive, the honeybee population is initialized according to Equation (1).

(1) $$\begin{array}{l} x_{i}^{j}=x_{min}^{j}+rand\;(0,1)\;(x_{max}^{j}-x_{min}^{j}) \end{array}$$

Where is the maximum value of the food source in the direction of *j*; $x_{\min}^{j}$ is the minimum value of the food source in the direction of *j*.

2. Employed bee stage: a solution is randomly selected from the external archive. The current domain search is guided according to Equation (2), and the better solution is retained through the Pareto dominance relation.

(2) $$\begin{array}{l} v_{i}^{j}=x_{i}^{j}+\phi_{i}^{j}\;(x_{i}^{j}-x_{k}^{j}) \end{array}$$

Where *x*_*k*_ denotes an adjacent food source,denotes the current food source, *k* ∈ {1, 2, ⋯ , *SN*} and *k* ≠ *i*. $\phi_{i}^{j}$ is a random number between [−1, 1].

3. Calculate the corresponding following probabilities for all solutions according to Equation (3).

(3) $$\begin{array}{l} P_{i}=\frac{{fit}_{i}}{\overset{SN}{\underset{j=1}{\sum }}{fit}_{j}}\\ {fit}_{i}=\frac{dom\;(i)}{SN} \end{array}$$

Where *fit*_*i*_ is the specific yield of the food source; *dom*(*i*) is the number of feasible solutions available in the total feasible solutions for the *i* and *SN* is the number of feasible solutions.

4. Onlooker bees stage: a solution is chosen randomly by the following probability, and then the domain search is performed using Equation (3).
5. Scout bees stage: the solution whose *trial* reaches the *Limit* is discarded and the food source information is initialized again according to Equation (1).
6. Update the external archive: add the Pareto optimal solution from the current population to the external archive and update it according to the crowding distance clipping when its upper limit is reached.
7. Export all solutions of external archive.

### 2.2. Multi-objective mobile robot path planning model

In this paper, we only consider the static known environment and assume that the route of the mobile robot from the starting point to the endpoint in the map environment is a series of path nodes.

Then the path of the mobile robot *Path* can be represented using *Path* = [*Start* = *P*_0_, *P*_1_, *P*_2_, ⋯ , *P*_*n*_, *P*_*n*+1_ = *End*]. Where *Start* and *End* are the starting point and the endpoint, respectively. *P*_*i*_ is the *i*th path node on the path and the two path nodes are considered to be connected by line segments. In this paper, two objective functions are proposed for the two performance metrics of path length and path safety, which are the path length function *f*_*l*_ and the path safety function *f*_*l*_. The mobile robot path planning problem is transformed into solving the optimal set of solutions of a multi-objective function, whose mathematical model is shown in Equation (4):


(4) $$\begin{array}{l} \{\begin{array}{l} F\;(Path)=\left[Start=P_{0},P_{1},P_{2},\cdots \;,P_{n},P_{n+1}=End\right]\\ Minimize\;f_{l}\;(Path)=Length\;(Path)\\ Minimize\;f_{s}\;(Path)=Safety\;(Path) \end{array} \end{array}$$

Satisfaction.


(5) $$\begin{array}{l} P_{0}=Start,\;P_{n+1}=End,\;Path\cap Obstacle=\emptyset \end{array}$$

That is, the path *Path* cannot intersect with an obstacle, but is allowed to coincide with the edge of the obstacle. Where represents the set of feasible paths, *Path* = {*Path*_1_, *Path*_2_, ..., *Path*_*m*_}.

In this paper, the adopted path safety function is defined as.


(6) $$\begin{array}{l} f_{s}=\{\begin{array}{l} f_{so},\;f_{so}\leq distance\\ const,\;f_{so}≻distance \end{array} \end{array}$$

Where *distance* represents the safe distance from the position of the obstacles in the working environment set in this paper; *Const* is a constant; *f*_*so*_ represents the shortest distance from the path of the mobile robot to all obstacles, and the definition of *f*_*so*_ is shown in Equation (7).


(7) $$\begin{array}{l} f_{so}=\overset{m}{\underset{k=1}{min}}\left\{Dist^{k}|Dist_{ij}^{k}=\sqrt{{\;(PathX_{i}^{k}-so_{j}^{x})}^{2}+{\;(PathY_{i}^{k}-so_{j}^{y})}^{2}}\right\} \end{array}$$

Where *i* = 1, 2, ⋯ , *n* represents the number of path nodes of each feasible path of the mobile robot; *j* = 1, 2, ⋯ , *r* is the number of obstacles in the working environment; *k* = 1, 2, ⋯ , *m* represents the number of feasible paths of the mobile robot; *Dist*^*k*^ represents the distance of the mobile robot path nodes in each feasible path from the obstacles in the working environment.

The total length of the path *Path* can be expressed by Equation (8).


(8) $$\begin{array}{l} L\;(Path)=\overset{n}{\underset{i=0}{\sum }}d\;(P_{i},P_{i+1}) \end{array}$$

Where *n* denotes the number of path nodes, *d*(*P*_*i*_, *P*_*i*+1_) denotes the distance between the point *P*_*i*_ and the point *P*_*i*+1_.

To further compare the performance advantages and disadvantages of the IMOABC algorithm in planning paths for mobile robots, path planning experiments were conducted with the single-objective artificial bee colony (ABC) algorithm under the same environmental map. The parameters in the ABC algorithm are set as follows: the bee colony size is 100 and the maximum number of iterations is 200. The cost equation is shown in Equation (9).


(9) $$\begin{array}{l} cost=\sqrt{{\;(X_{g}-X_{i})}^{2}+{\;(Y_{g}+Y_{i})}^{2}}\\ \;+\;(collide*5000+oldpoint\text{\_}cost+collide\text{\_}to\text{\_}target)\\ \;*0.5*sqrt\;(MM\hat{\;}2+MM\hat{\;}2) \end{array}$$

Where *X*_*i*_, *Y*_*i*_ are the coordinates of the food source and *X*_*g*_, *Y*_*g*_ are the coordinates of the target point; *oldpoint*_*cost* is the value of the previously selected path; *collide*_*to*_*target* has two cases: Case 1: When a collision occurs, the collision-to-target value is equal to zero, and Case 2: When there is no collision, the collision-to-target value is equal to one.

### 2.3. Performance metrics for mobile robot path planning

This thesis evaluates the goodness of the feasible path obtained by the multi-objective optimization algorithm by the length of the path obtained by the optimization algorithm and the path safety.

#### 2.3.1. Criteria for measuring path length

Among the many performance metrics for path planning, only the length size of the feasible path of the mobile robot optimized by the multi-objective optimization algorithm is considered. A long path is inferior to a short path.

#### 2.3.2. Path safety metrics

Among the many performance metrics of path planning, only the path safety of the mobile robot optimized by the multi-objective optimization algorithm is considered. A path with a small path safety value is inferior to a path with a large path safety value. The formula for calculating the safety indicator for each route of the mobile robot is shown in Equation (10).


(10) $$\begin{array}{l} S=min\left\{Dist|Dist_{ij}=\sqrt{{\;(PathX_{i}-so_{j}^{x})}^{2}+{\;(PathY_{i}-so_{j}^{y})}^{2}}\right\} \end{array}$$

Where *i* = 1, 2, ⋯ , *n*, represents the number of path nodes; *j* = 1, 2, ⋯ , *r*, represents the number of obstacles in the working map environment.

## 3. Improvement and validation of MOABC algorithm

### 3.1. Improvement of MOABC algorithm

While the MOABC algorithm has many advantages, it also has its disadvantages. First, since there is a greater demand for external archive space, the algorithm will have higher requirements for the objective environment. Second, although the algorithm takes all objective functions into account when optimizing, the degree of optimization of them will still be different in practical applications, which will make the results with a certain bias. Third, when solving multi-objective problems, the algorithm suffers from problems such as the tendency to fall into local optimality. In this paper, we propose an improved MOABC algorithm, namely IMOABC algorithm, for a series of problems existing in the above MOABC algorithm. Inspired by the ideas of algorithms such as NSGA-II and DNPSO, this paper combines the MOABC algorithm with a series of multi-objective optimization strategies (Deb et al., 2002; Zhou et al., 2017). It consists of four important strategies: the external archive pruning strategy, the non-dominated ranking and crowding distance strategy, the search strategy, and the Pareto optimal strategy to evaluate food source locations with multiple objectives and select non-dominated solutions, and is thus presented as follows.

#### 3.1.1. External archive pruning strategy

**Figure 1:**
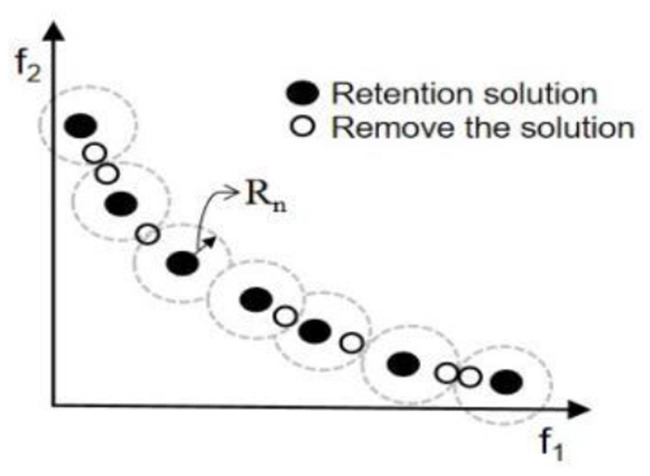
Schematic diagram of external file trimming.

To reduce the complexity of the algorithm computation and to obtain a uniformly distributed solution, the size of the external archive should not increase rapidly. Therefore, to prune external archive, this paper uses a technique based on the concept of domain radius for pruning external archive. The domain radius (Rn) of the non-dominated solution is considered and the Euclidean distance between the non-dominated solutions is calculated at the end of each iteration. If the distance between two non-dominated solutions is less than Rn, one of the non-dominated solutions is omitted. Figure 1 illustrates this process in the space of two objective functions.

#### 3.1.2. Non-dominated sorting and congestion distance strategies

Inspired by the ideas of the NSGA-II algorithm, in this thesis we also introduce the concepts of fast non-dominated sorting and congestion distance (Deb et al., 2002). The individuals in the population that are not dominated by non-inferior solutions are defined as rank 1, then they are removed from the population and new non-inferior solutions are identified from the remaining individuals and defined as rank 2; the above process was repeated until all individuals in the population were given the appropriate rank. By storing the current solution and all individuals in the population in a hierarchical manner, the top-performing individuals can have a higher probability of survival and can quickly improve the population ranking.

**Figure 2:**
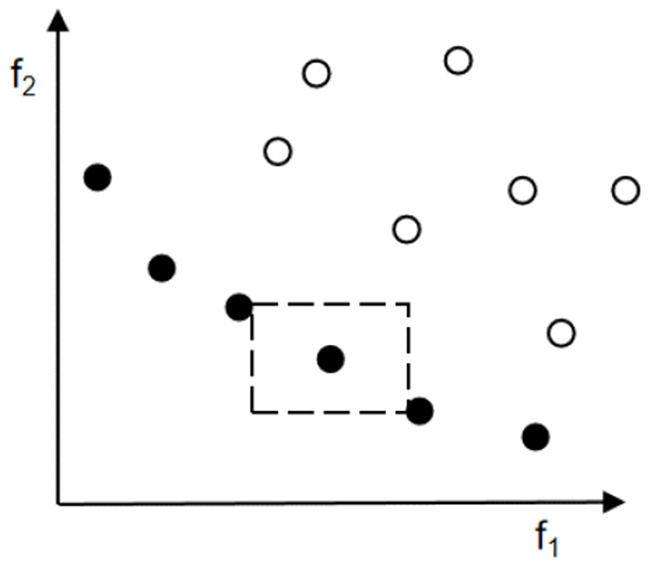
Diagram of congestion distance.

Once the number of non-inferior solutions exceeds the external archive size, the removal of individuals with small crowding distance in the external archive starts until the algorithm condition is satisfied (Akbari et al., 2012). As shown in Figure 2, regarding the two objective problems, the black dots in the figure are the non-inferior solutions in the population, and for the non-inferior solution *x*, the sum of the two sides of the rectangle consisting of *x* + 1 and *x* − 1 is computed; the final result is the crowding distance *x*_*dist*_ of the non-inferior solution *x*. The crowding distance of the boundary solutions (solutions with minimum and maximum objective function values) is infinite. When two solutions have the same rank, the superiority of the two solutions can be compared by the magnitude of the crowding distance, thus ensuring the convergence and diversity of the population. It can be seen that the individual *x* is superior to the individual *y* when and only when, *x*_*rank*_ < *y*_*rank*_ or *x*_*rank*_ = *y*_*rank*_ and *x*_*dist*_ > *y*_*dist*_.

#### 3.1.3. Pareto optimal strategy

The Pareto solution set is a variety of feasible solutions for mobile robot path planning, which can also improve the efficiency of mobile robot path planning. Manual Pareto selection is subject to various subjective uncertainties, which can make the chosen solution not optimal. Fix a function η_*i*_ and the function η_*i*_ is denoted as


(11) $$\begin{array}{l} \eta_{i}=\{\begin{array}{l} 1,\;V_{i}\leq V_{i}^{min}\\ \frac{V_{i}^{max}-V_{i}}{V_{i}^{max}-V_{i}^{min}},\;V_{i}^{min}\leq V_{i}\leq V_{i}^{max}\\ 0,\;V_{i}\geq V_{i}^{max} \end{array} \end{array}$$

Where $V_{i}^{max}$ and $V_{i}^{min}$ are the maximum and minimum values of the *i*th objective function in the Pareto solution set, respectively; *V*_*i*_ is the *i*th objective function value. For each non-inferior solution *k*, the normalized affiliation function η_*k*_ is calculated by the following equation.


(12) $$\begin{array}{l} \eta_{k}=\frac{\sum_{i=1}^{Nob}\eta_{i}^{k}}{\sum_{j=1}^{M}\sum_{i=1}^{Nob}\eta_{i}^{j}} \end{array}$$

Where *M* is the number of non-inferior solutions in the Pareto solution set and *N*_*ob*_ is the number of objective functions. The larger the value of η_*k*_, the better the performance *k* of in coordinating multiple objective functions. The ranking of the Pareto solution set by the value of η_*k*_ gives the priority sequence of non-inferior solutions.

#### 3.1.4. Search strategy

As one of the most representative variants, the DNPSO algorithm is characterized by the introduction of the global *NS* operator. Therefore, the search strategy in this paper also adds that operator, which can be expressed as:


(13) $$\begin{array}{l} v_{i}^{j}=r_{1}x_{i}^{j}+r_{2}x_{gbest}+r_{3}\;(x_{a}^{j}-x_{b}^{j}) \end{array}$$

**Figure 3:**
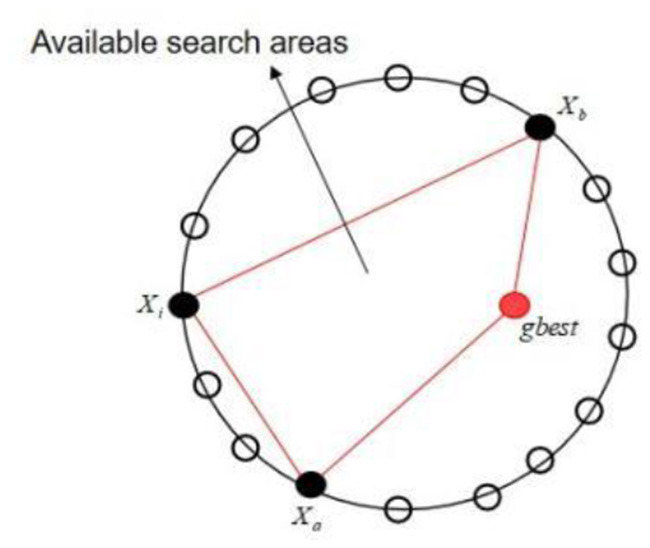
Global domain search operator.

Where *r*_1_, *r*_2_ and *r*_3_ are three mutually exclusive numbers chosen at random from (0, 1), which must satisfy another condition: *r*_1_ + *r*_2_ + *r*_3_ = 1; *x*_*gbest*_ is the global optimal solution for the whole population; and the indices *a* and *b* are mutually exclusive integers chosen at random from (1, 2, ⋯ , *SN*), which are different from the basic index *i*. It is important to note that *r*_1_, *r*_2_ and *r*_3_ are regenerated in each generation, but they remain the same for all dimensions of each generation. Once an experimental solution has been generated, its associated food source must compete with it for entry into the next generation. This means that individuals with better fitness values have a chance to survive. An explicit illustration of the global *NS* operator is shown in Figure 3.

Thus, the basic flow of the IMOABC algorithm is shown below.

Step 1: Population initialization, randomly generate *SN* feasible solutions according to equation (1).
Step 2: Calculate the objective function values for all individuals and complete the initialization of the external archive set.
Step 3: In the employed bees stage, the employed bees guide the current domain search according to Equation (13).
Step 4: During the onlooker bees stage, the onlooker bees randomly select the nectar source according to Equation (3).
Step 5: Add all non-dominated solutions of the current population to the external archive according to the external archive pruning strategy.
Step 6: During the scout bees stage, a nectar source is randomly selected according to Equation (1).
Step 7: When the number of non-inferior solutions exceeds the *limit* number of external archives, fast non-dominated sorting is performed on solutions in external archives using the grouping of solutions and the
Step 8: Determine if the termination condition is met. If yes, output the Pareto front; otherwise go to step 3.
Step 9: The set of Pareto solutions is sorted by the Pareto optimal strategy, and then a priority list of solutions is derived.

### 3.2. Performance testing and analysis of an IMOABC algorithm

#### 3.2.1. Test functions and performance metrics

To test the IMOABC algorithm proposed in this paper, simulation tests will be carried out for the single-objective optimization problem. In this paper, six standard test functions UF1-UF6 are selected, and all six test functions have two optimization objectives. The inverse generation distance (IGD) is chosen as the evaluation criterion to compare the performance of the algorithms. The inverse generation distance (IGD) is used to evaluate the comprehensive performance of the algorithm (convergence and distributivity) and is defined as (Durillo and Nebro, 2011).


(14) $$\begin{array}{l} IGD\;(P,\;Q)=\frac{\sum_{v\in P}d\;(v,Q)}{\left|P\right|} \end{array}$$

Where *P* is the set of points on the real Pareto front end and |*P*| is the number of *P*. *Q* is the set of Pareto optimal solutions. And *d* (*v, Q*) is the minimum Euclidean distance from the individual to the population *Q* in *P*. The smaller the value, the better the overall performance of the algorithm.

#### 3.2.2. Test function simulation results and analysis

**Figure 4:**
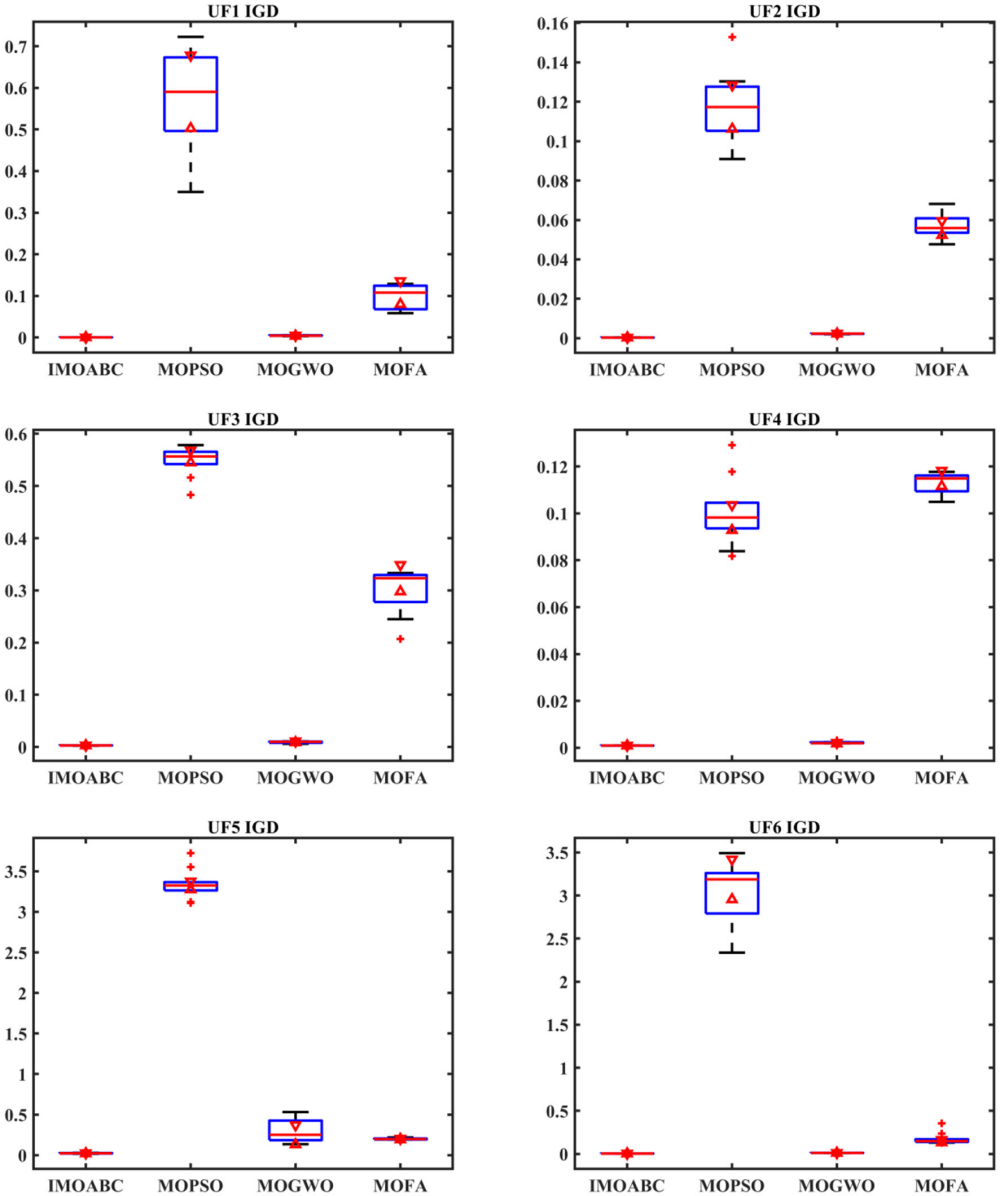
Test function IGD boxplot.

To test the performance of the IMOABC algorithm proposed in this paper, three typical multi-objective algorithms will be selected for comparison, i.e., they will be compared with MOPSO, MOGWO, and MOFA. To ensure the fairness of the algorithm testing, the parameter settings for each algorithm are given in Table 1, and the algorithms are run 20 times separately and independently to reduce any unexpected errors in the algorithm's operation. By counting and comparing the maximum value (Max), minimum value (Min), mean value (Mean), and standard deviation (Std) of IGD for these four algorithms, the results are shown in Table 2, where the bolded items are the optimal values. At the same time, to have a better feeling of the performance comparison between each algorithm, we introduce box plots, which can more intuitively show the dispersion of the performance index data between each algorithm. We then plotted the values of the IGD metrics in Table 2 separately into the box plots shown in Figure 4.

**Table 1:** Set each algorithm parameter.

**Algorithm**	**Parameter setting**
IMOABC	SN = 100 Limit = 100 maxCycle = 5,000 runtimes = 20
MOPSO	SN = 100 Limit = 100 maxCycle = 5,000 runtimes = 20 alpha = 0.1 beta = 4 gamma = 2
MOGWO	SN = 100 Limit = 100 maxCycle = 5,000 runtimes = 20 alpha = 0.1 beta = 4 gamma = 2
MOFA	SN = 100 Limit = 100 maxCycle = 5,000 runtimes =2 0
	Gamma = 2 beta 0 = 1 alpha = 0.1 alpha_damp = 0.9 mu = 0.1

**Table 2:** Statistical results of IGD value of 6 test functions of each algorithm.

**Function**		**IMOABC**	**MOPSO**	**MOGWO**	**MOFA**
UF1	Max	6.0977E-04	7.2238E-01	5.6043E-03	1.2877E-01
	Min	3.0968E-04	3.4987E-01	3.4772E-03	5.8557E-02
	Mean	3.9535E-04	5.8111E-01	4.5354E-03	9.6393E-02
	Std	9.8998E-05	1.1530E-01	6.8383E-04	2.6902E-02
UF2	Max	3.9503E-04	1.5286E-01	2.6193E-03	6.8151E-02
	Min	2.7110E-04	9.0947E-02	2.0654E-03	4.7688E-02
	Mean	3.1956E-04	1.1721E-01	2.2934E-03	5.7197E-02
	Std	4.0266E-05	1.6970E-02	1.4827E-04	6.3076E-03
UF3	Max	3.7099E-03	5.7834E-01	1.0777E-02	3.3310E-01
	Min	2.4375E-03	4.8281E-01	6.1589E-03	2.0697E-01
	Mean	2.9466E-03	5.4727E-01	9.2236E-03	3.0021E-01
	Std	3.3504E-04	2.6733E-02	1.5362E-03	4.1251E-02
UF4	Max	1.0123E-03	1.2913E-01	3.1823E-03	1.1771E-01
	Min	8.9674E-04	8.1743E-02	1.9092E-03	1.0491E-01
	Mean	9.4301E-04	1.0063E-01	2.1780E-03	1.1295E-01
	Std	3.8383E-05	1.3554E-02	3.7717E-04	3.9907E-03
UF5	Max	3.3773E-02	3.7239E+00	5.3183E-01	2.3188E-01
	Min	1.7787E-02	3.1088E+00	1.3546E-01	1.9165E-01
	Mean	2.3999E-02	3.3441E+00	2.9843E-01	2.0271E-01
	Std	4.6789E-03	1.7392E-01	1.3587E-01	1.2418E-02
UF6	Max	6.7739E-03	3.4929E+00	1.7238E-02	3.5559E-01
	Min	2.1384E-03	2.3373E+00	1.0387E-02	1.3182E-01
	Mean	4.3481E-03	3.0233E+00	1.2353E-02	1.7620E-01
	Std	1.3624E-03	3.4637E-01	1.8345E-03	6.6398E-02

From the IGD of the integrated performance metrics in Table 2 and Figure 4, it can be seen that for these six test functions, the IMOABC algorithm outperforms the other three algorithms compared to the other three algorithms, and its IGD data values of integrated performance metrics are more concentrated. This shows that the IMOABC algorithm is also more stable in solving multi-objective optimization problems, further demonstrating the effectiveness of the IMOABC algorithm.

**Figure 5:**
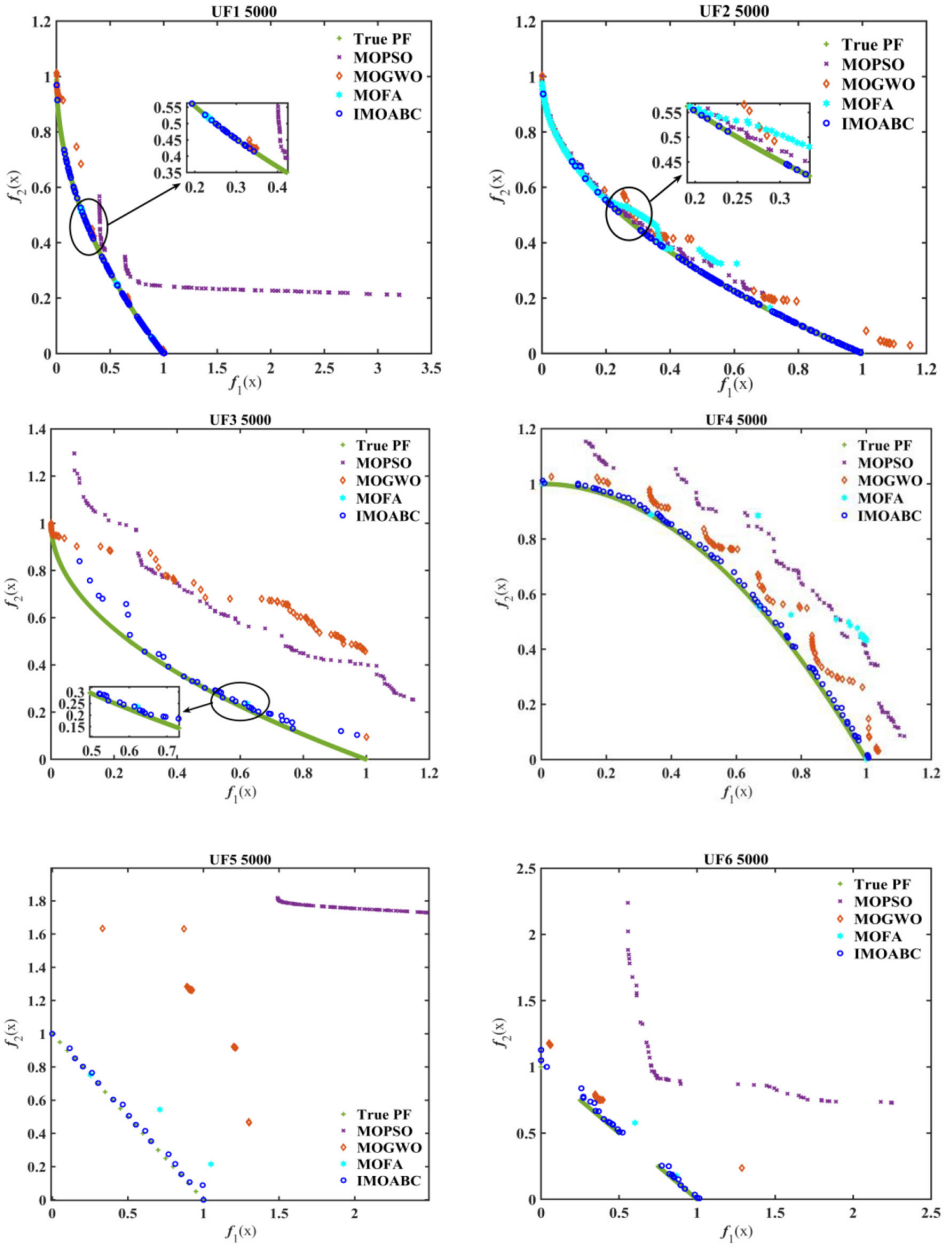
Test function Pareto frontier comparison graph.

To visually and compare the performance of each algorithm, Figure 5 gives a comparative plot of the Pareto frontier for these four algorithms solving the six standard test functions.

From Figure 5 we can see more intuitively that the approximate Pareto front obtained by the IMOABC algorithm is closer to the real Pareto front than the other three algorithms. In particular, for the UF5 problem, the IMOABC algorithm shows a large improvement in the combined performance metrics compared to the remaining three algorithms. This further demonstrates that the IMOABC algorithm proposed in this paper not only yields a better approximation of the frontier, but also its convergence and distributivity metrics are superior and have been improved.

## 4. Simulation verification

### 4.1. Establishment of mobile robot optimization environment

Before a mobile robot searches for a path, it is important to first model the workspace in which it is located to facilitate identification and decision-making by the mobile robot. Environment modeling refers to the formal description of the realistic environment of a mobile robot through feature analysis and then replacing the obstacle information into a language that the computer can understand using a suitable approach. A variety of methods for environment modeling exist in the world, and the three most basic and commonly used methods are the raster method, the geometric feature method, and the topological method (Zhang et al., 2018).

**Figure 6:**
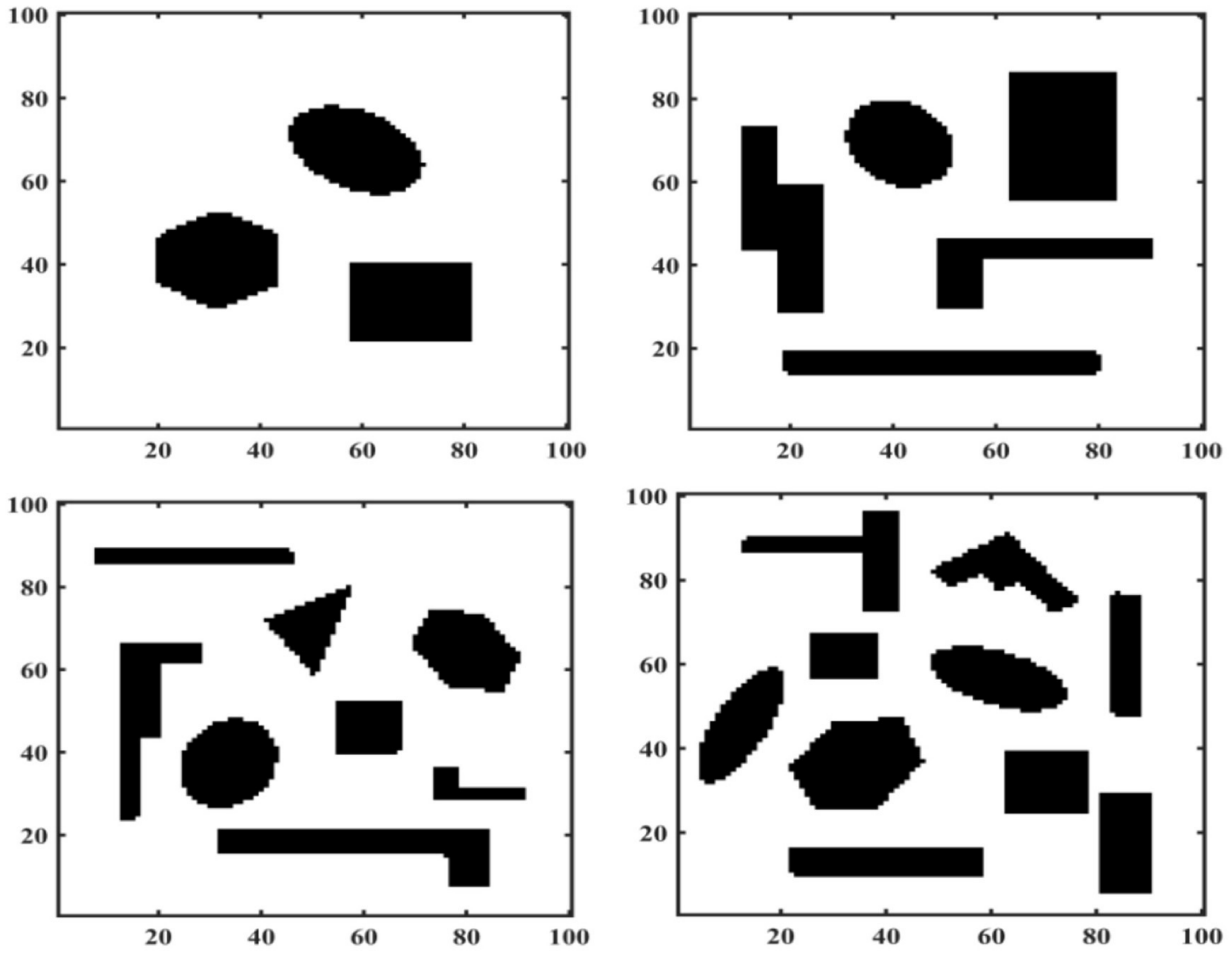
Mobile robot working environment.

There are advantages and disadvantages to each of these three approaches to constructing a work environment map. The object of this paper is mainly for land mobile robots, whose working environment is a two-dimensional plane, so the motion space of mobile robots is represented by a two-dimensional coordinate system. The mobile robot is represented by a mass point, and an image in BMP format is set up as the actual working environment of the mobile robot. The size of this BMP is 100^*^100, the black part is the obstacle area and the white part is the feasible area. The obstacle locations and shapes in the four different BMPs are shown in Figure 6. The obstacle shapes in the BMPs are set to geometric polygons to facilitate verification of the effectiveness of IMOABC algorithm-based path planning in complex and variable environments.

### 4.2. Experimental simulations and algorithm comparison

The effectiveness of the IMOABC algorithm proposed in this thesis has been confirmed in the previous section, so this section will explore the application of the IMOABC algorithm to the mobile robot path planning problem and verify its effectiveness. Here, we have conducted simulation experiments using Matlab 2018b, and the two selected objective functions are path length and path safety. Firstly, the effectiveness of the IMOABC algorithm applied to mobile robot path planning in a two-dimensional working environment with complex and variable obstacles is verified, followed by a comparative analysis with path planning based on the MOABC algorithm and ABC algorithm. Also, to verify the robustness of the algorithm, two experiments were conducted in the same experimental environment by changing the starting and ending coordinates of the mobile robot path planning, with the starting coordinates of case 1 being (0, 0) and the ending coordinates being (100, 100); the starting coordinates of case 2 being (0, 100) and the ending coordinates being (100, 0).

#### 4.2.1. Simulation and verification for case 1

**Figure 7:**
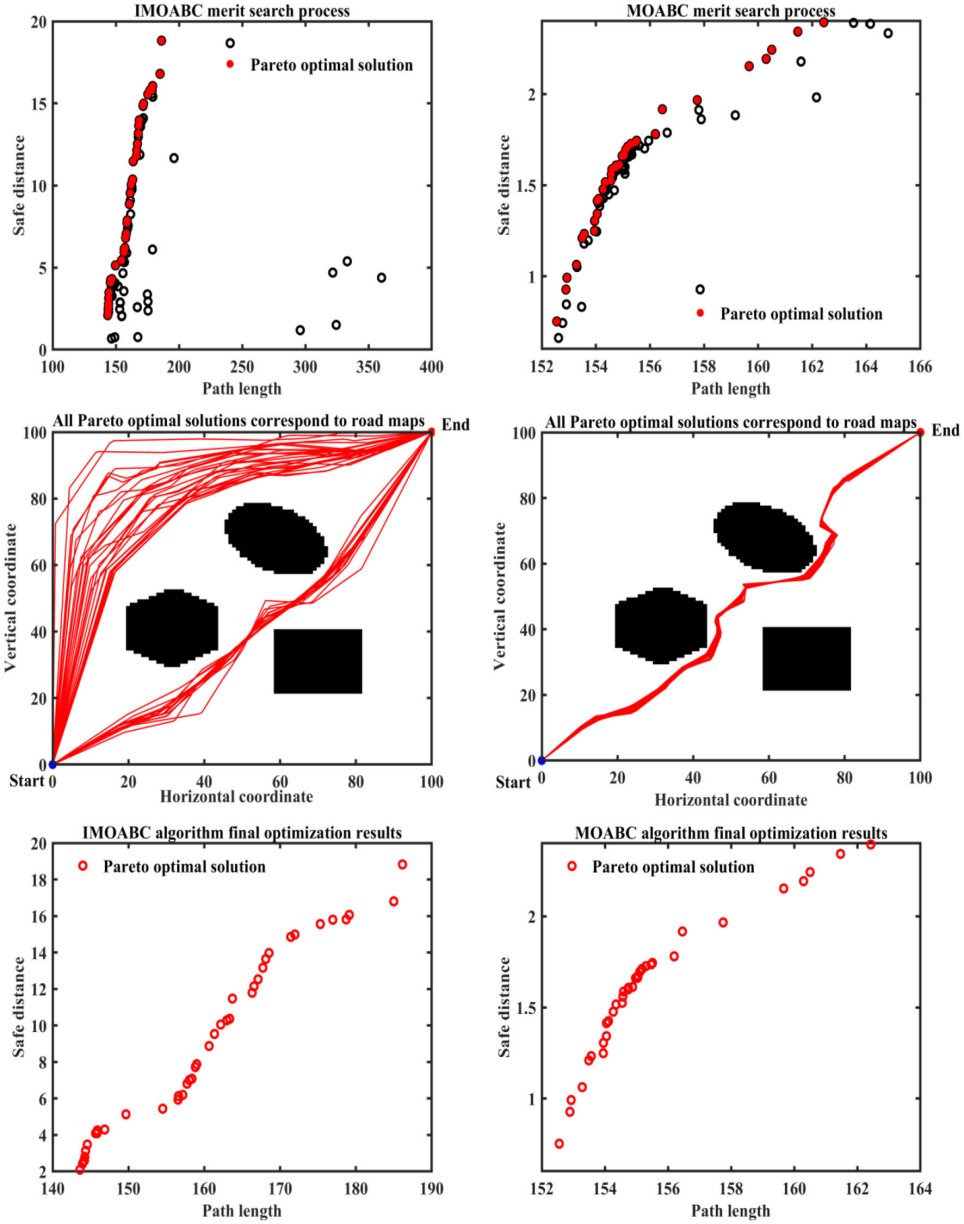
Experimental results of path planning based on IMOABC algorithm and MOABC algorithm in map 1 for case 1.

**Figure 8:**
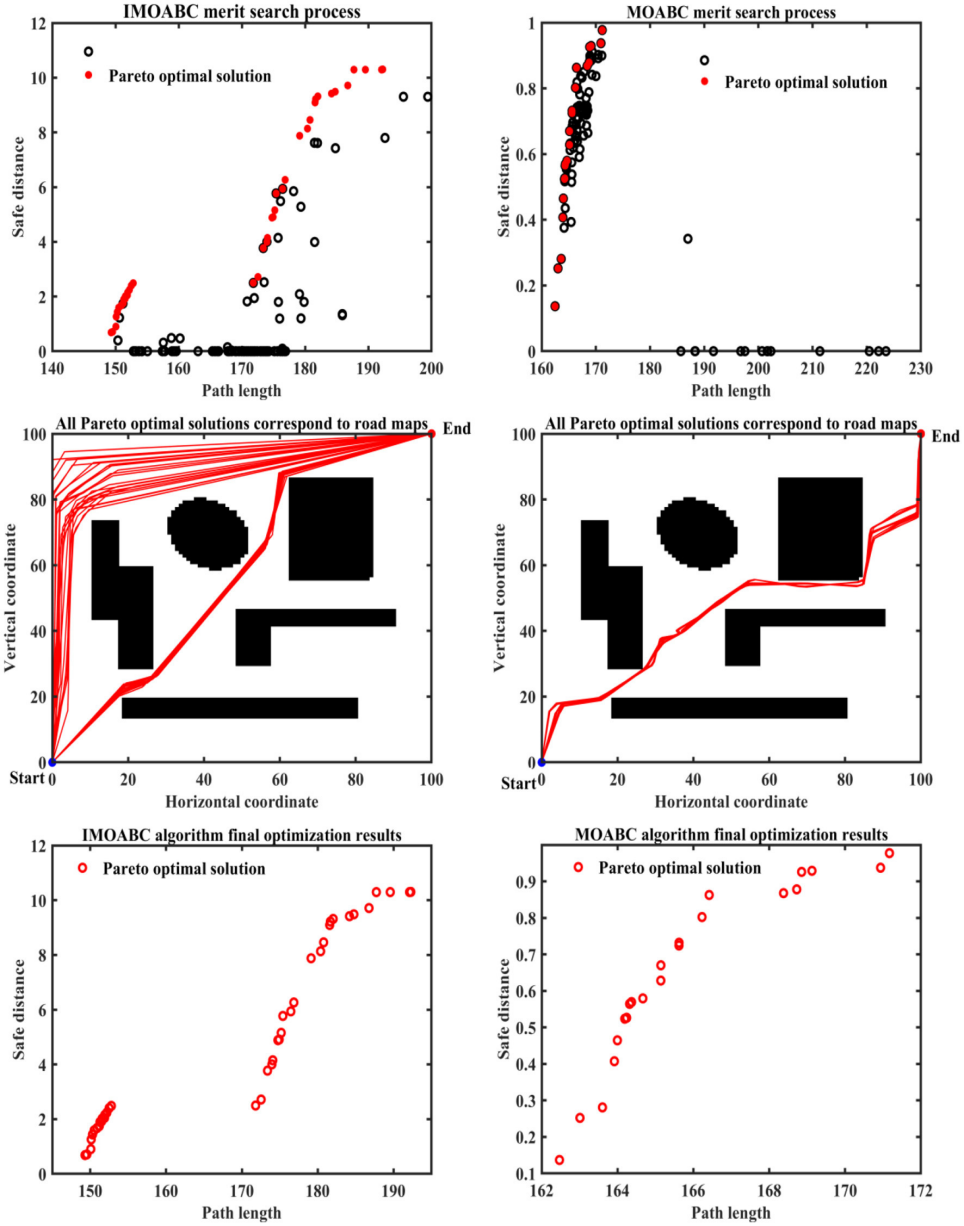
Experimental results of path planning based on IMOABC algorithm and MOABC algorithm in map 2 for case 1.

**Figure 9:**
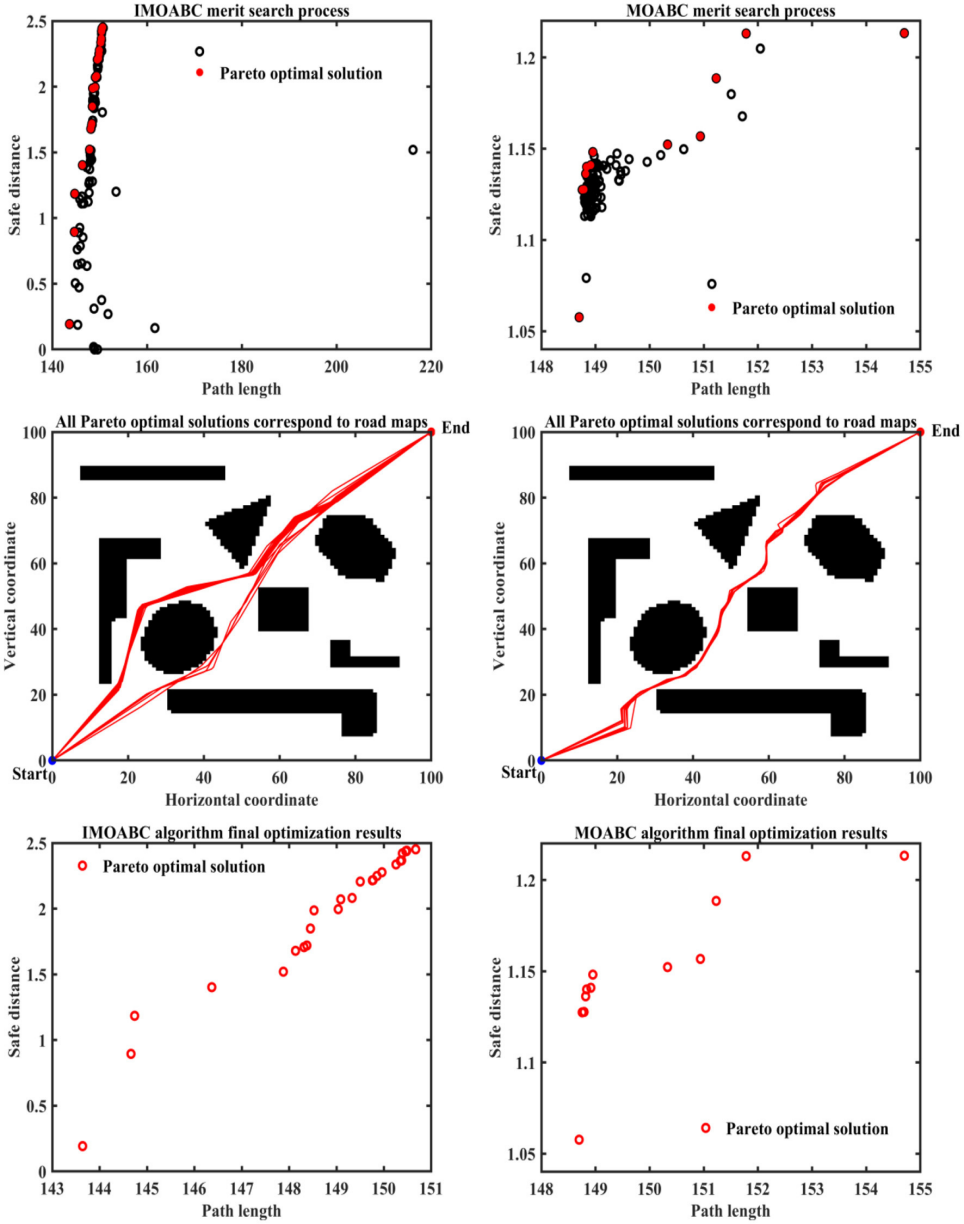
Experimental results of path planning based on IMOABC algorithm and MOABC algorithm in map 3 for case 1.

**Figure 10:**
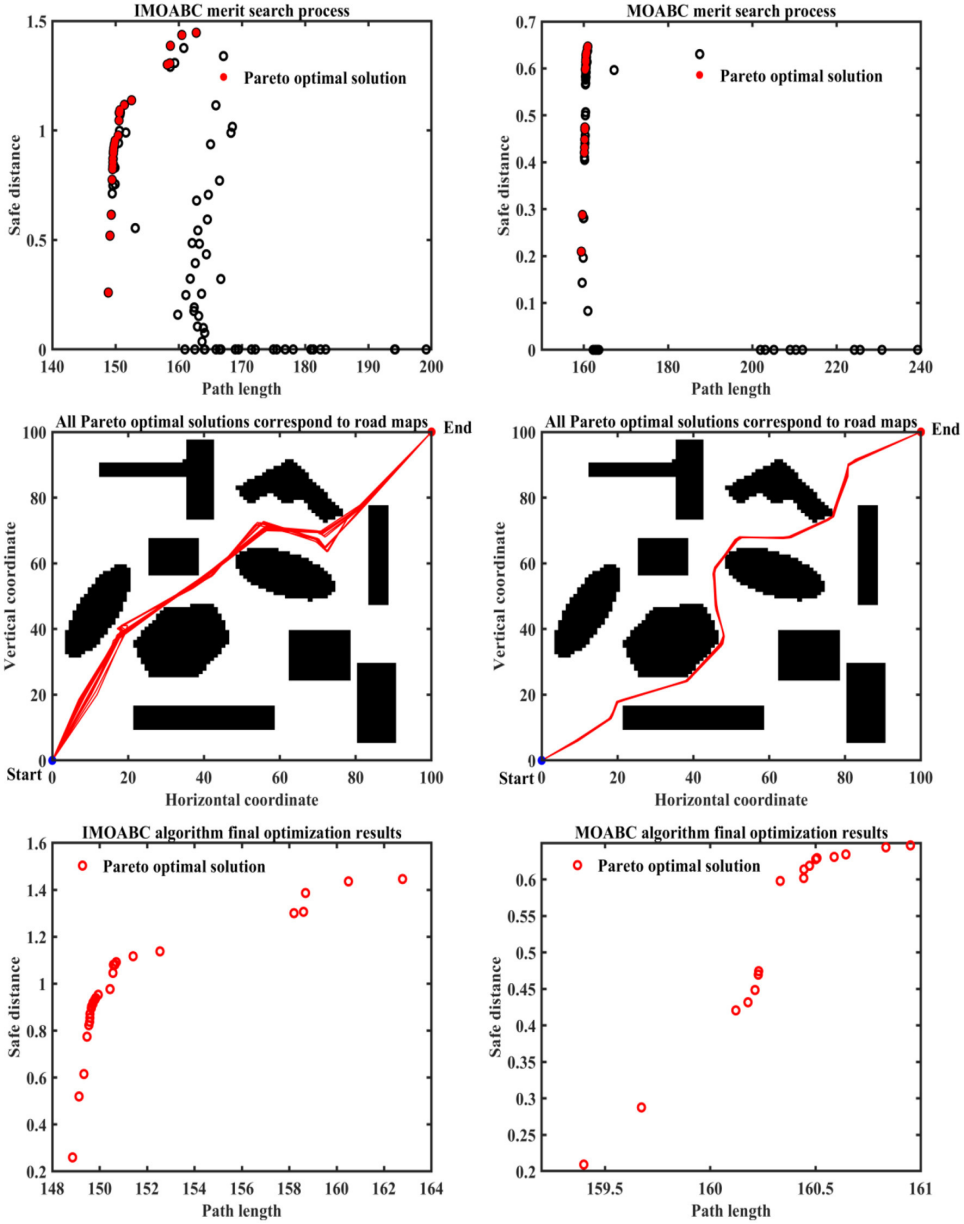
Experimental results of path planning based on IMOABC algorithm and MOABC algorithm in map 4 for case 1.

**Figure 11:**
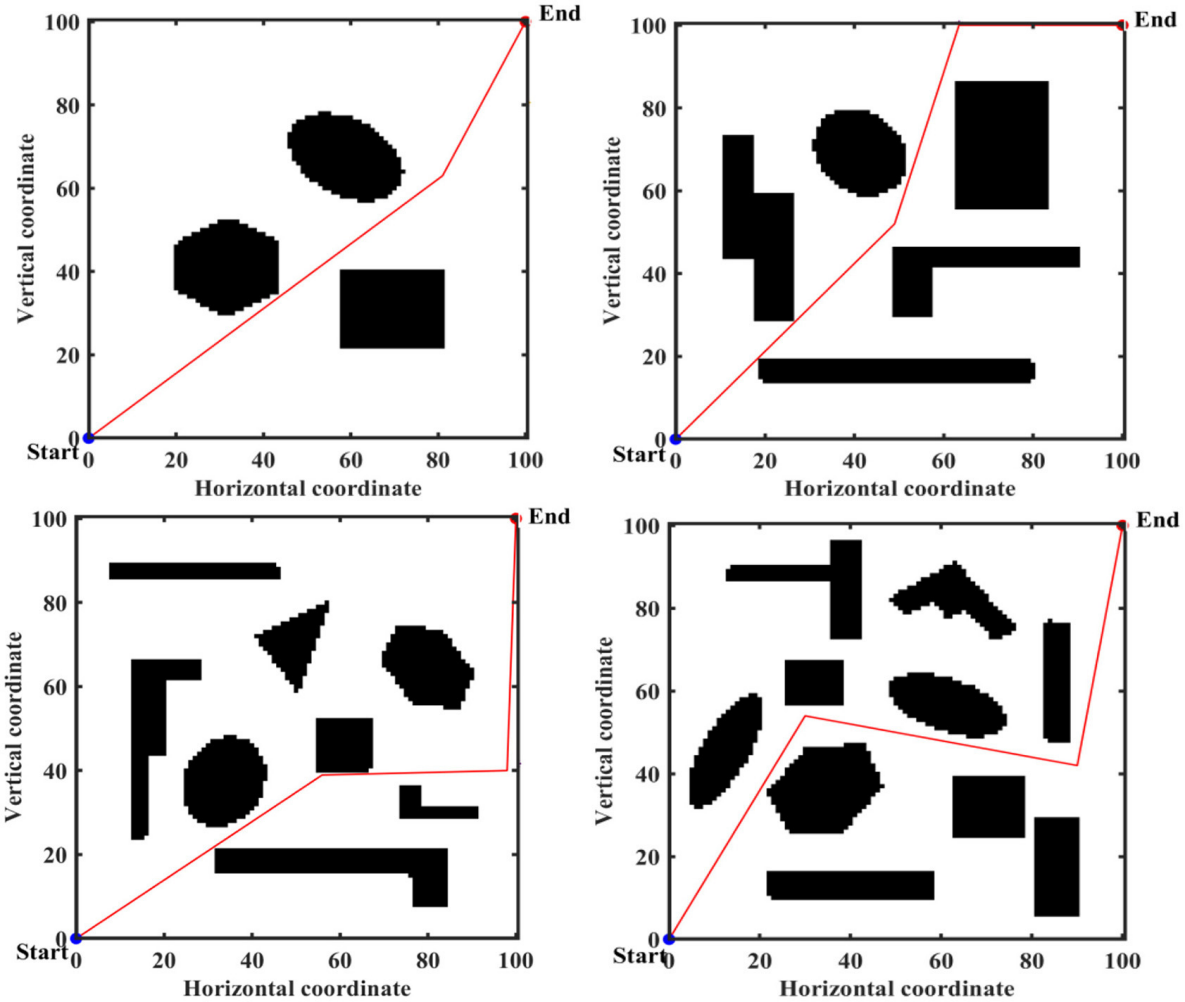
Path planning results of mobile robot in 4 different environments based on ABC algorithm for case 1.

To verify the effectiveness of IMOABC algorithm-based path planning, path planning experiments are first conducted in four environments with different obstacles. The initialization algorithm parameters are: the bee colony size is set to 100, the maximum number of iterations of the algorithm is set to 200, the four different 2D working environment pixels constructed using BMPs are uniformly set to 100^*^100, and the starting coordinates of the experimentally set path planning are (0, 0) and the ending coordinates are (100, 100). The results are shown in Figures 7–11, where the mobile robot harvested several feasible routes. Mobile robots can select routes from planning results based on environmental conditions and their preferences. As can be seen, the mobile robot can perfectly avoid complex and variable obstacles and find the minimum path distance from the starting point to the endpoint.

From Table 3 and Figures 7–11 we can find that the path length of the mobile robot based on the IMOABC algorithm is the shortest in all four environments. That is, the path length performance of path planning based on the IMOABC algorithm is better than the path length performance of path planning based on the MOABC algorithm and ABC algorithm.

**Table 3:** The first experiment is to compare the path planning data between each algorithm.

**Performance indicators**	**Working environment**		**IMOABC**	**MOABC**	**ABC**
Path length	Map 1	Max	186.144	162.428	**144.2091**
		Min	**143.605**	152.553	144.2091
	Map 2	Max	192.274	171.17	**158.1544**
		Min	**149.317**	162.469	158.1544
	Map 3	Max	**150.671**	154.703	170.2625
		Min	**143.641**	148.697	170.2625
	Map 4	Max	162.777	**160.95**	181.8178
		Min	**148.855**	159.4	181.8178
Path security	Map 1	Max	**18.827**	2.39384	0.588
		Min	**2.09754**	0.752478	0.588
	Map 2	Max	**10.3018**	0.977297	0.0868
		Min	**0.686048**	0.136834	0.0868
	Map 3	Max	**2.45146**	1.21333	0.238
		Min	0.19272	**1.05764**	0.238
	Map 4	Max	**1.44622**	0.646673	0.996
		Min	0.259739	0.209394	**0.996**
Number of possible routes	Map 1		42	36	1
	Map 2		43	11	1
	Map 3		26	12	1
	Map 4		27	17	1

The meaning of the bold values is the optimal value.

From Table 3 and Figures 7–11 we can find that the path safety of the IMOABC algorithm-based path planning is maximum in the map 1 and map 2 environments. That is, the path safety of path planning based on the IMOABC algorithm is better than the path safety performance of both MOABC algorithm-based and ABC algorithm-based path planning. In the map 3 environment, we can find from Figure 9 that the safety performance of path planning based on the IMOABC algorithm is the most superior. In the map 4 environment, the path safety of path planning based on the IMOABC algorithm is higher than the path safety of path planning based on the MOABC algorithm and slightly inferior to the path safety of path planning based on the ABC algorithm when the path is shortest, but the shortest path of path planning based on ABC algorithm is much inferior to the shortest path of path planning based on IMOABC algorithm. In summary, it is still the IMOABC algorithm-based path planning that has the best safety performance.

From Table 3 and Figures 7–11 we can find that the number of feasible routes for path planning based on the IMOABC algorithm is more than that of the MOABC-based algorithm and ABC-based algorithm in these four environments. And the IMOABC algorithm-based path planning feasible routes are a little more dispersed than the MOABC algorithm and ABC algorithm-based path planning feasible routes, which means that the IMOABC algorithm-based path planning has more options in choosing feasible routes according to their situation. None of the path planning based on the MOABC algorithm in the map 2 environment can completely draw the number of feasible routes. Therefore, the IMOABC algorithm-based path planning for mobile robots is the most superior in terms of the number of feasible routes.

In summary, the experimental results demonstrate the superiority of IMOABC algorithm-based path planning for mobile robots in terms of three aspects: path length, path safety, and the number of feasible routes.

#### 4.2.2. Simulation and verification for case 2

To verify the robustness of the IMOABC algorithm applied to the mobile robot, we conduct a second experiment on the mobile robot, where the starting coordinates are set to (0, 100) and the ending coordinates are (100, 0). The results of the path planning experiment are as follows.

**Figure 12:**
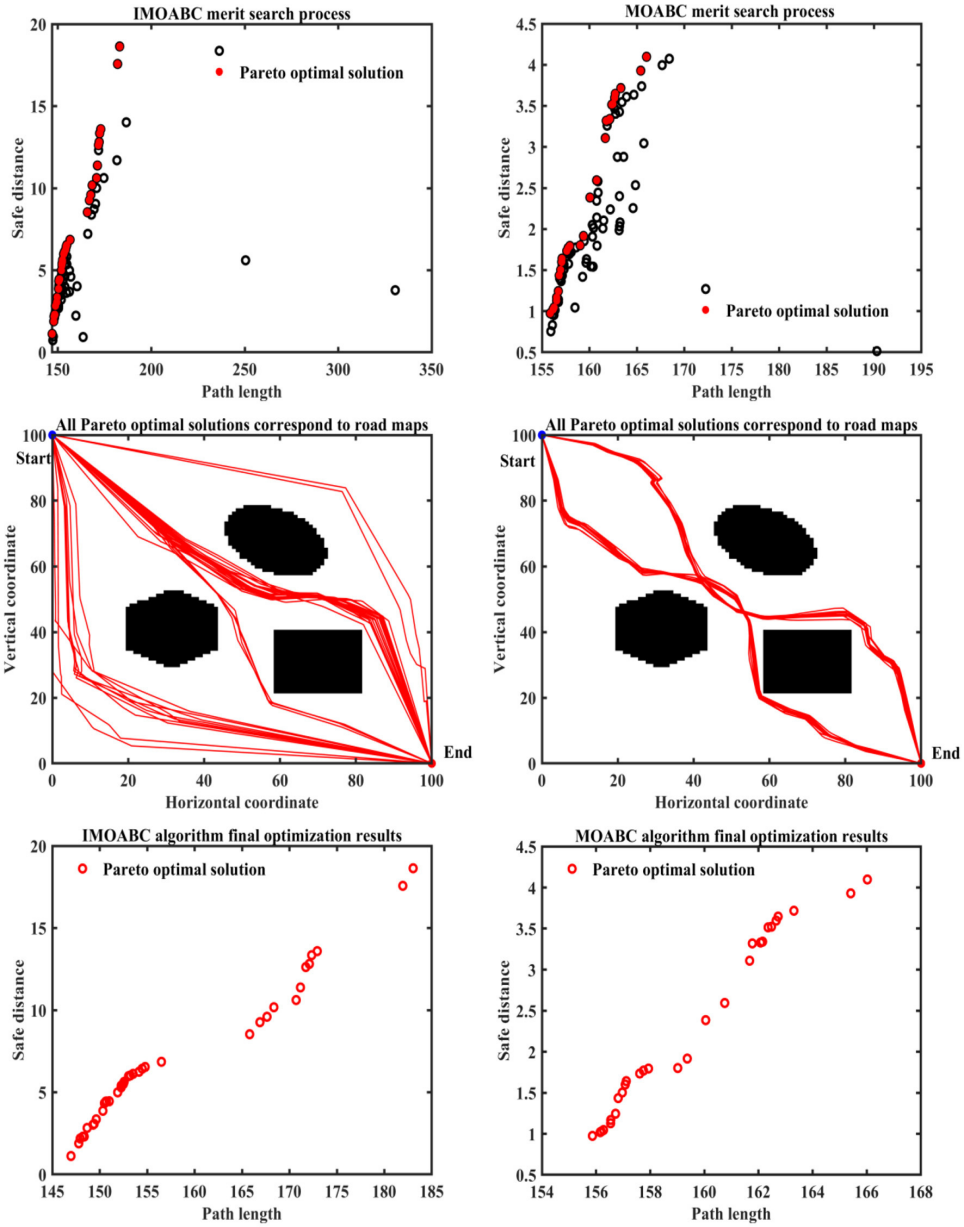
Experimental results of path planning based on IMOABC algorithm and MOABC algorithm in map 1 for case 2.

**Figure 13:**
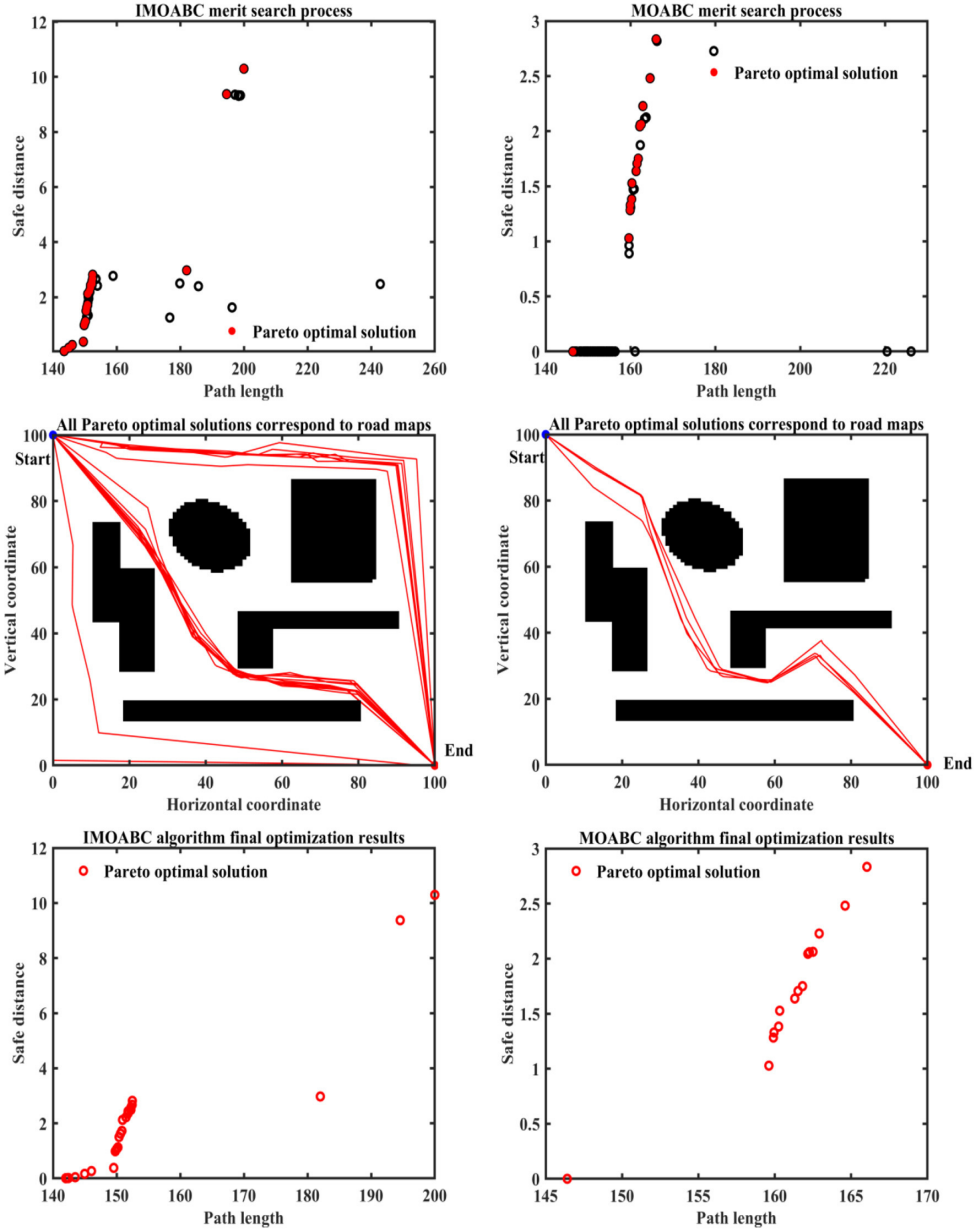
Experimental results of path planning based on IMOABC algorithm and MOABC algorithm in map 1 for case 2.

**Figure 14:**
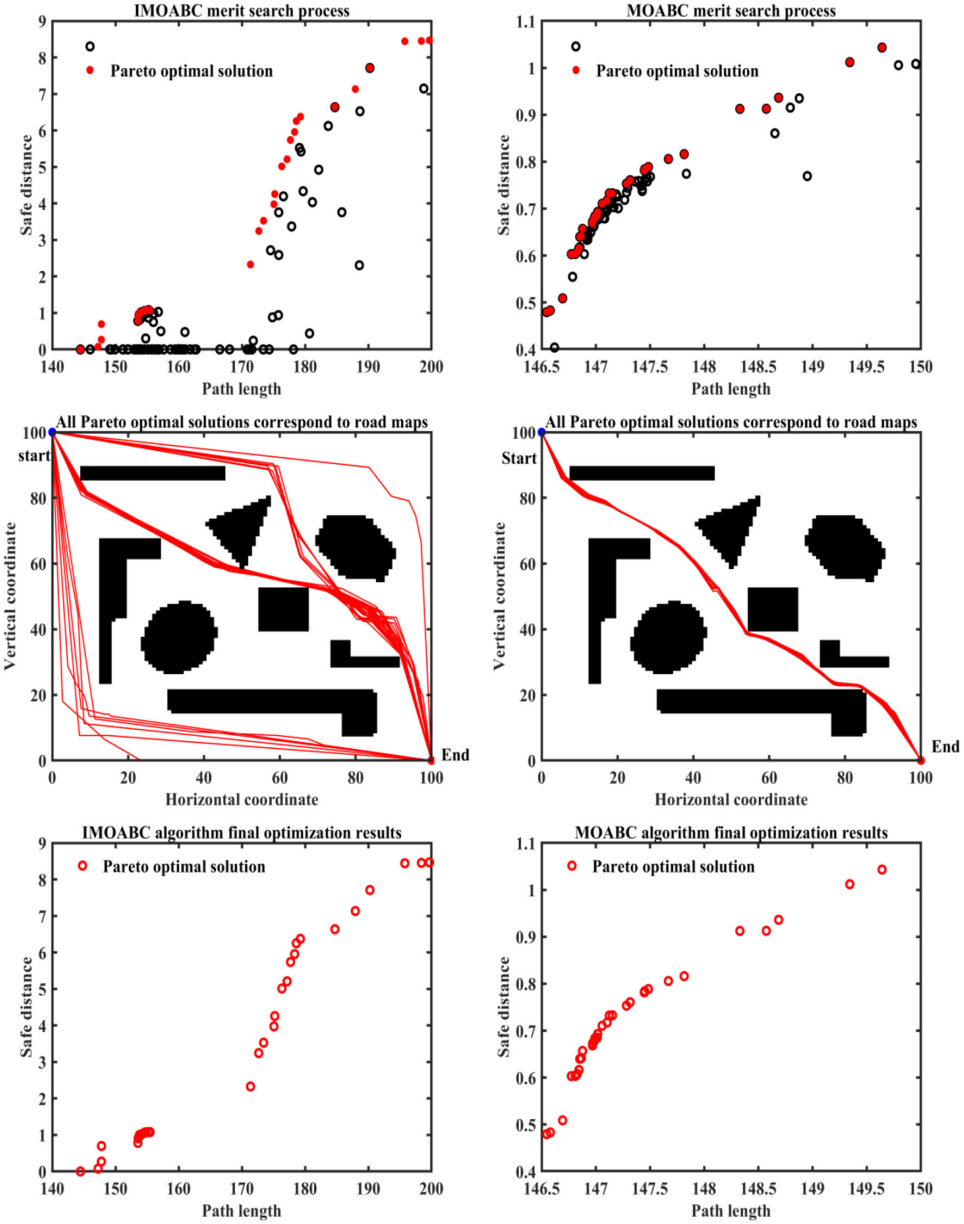
Experimental results of path planning based on IMOABC algorithm and MOABC algorithm in map 3 for case 2.

**Figure 15:**
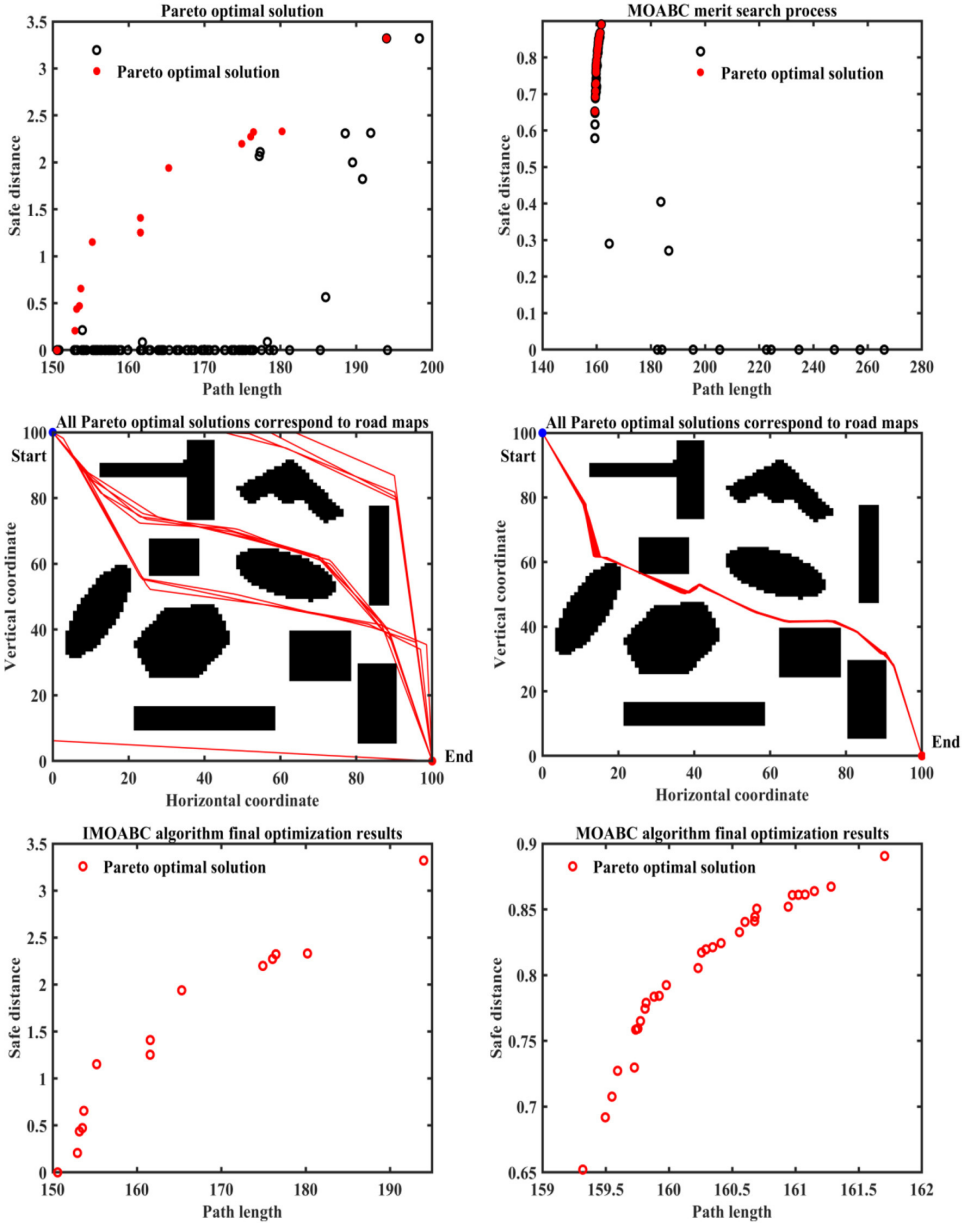
Experimental results of path planning based on IMOABC algorithm and MOABC algorithm in map 4 for case 2.

**Figure 16:**
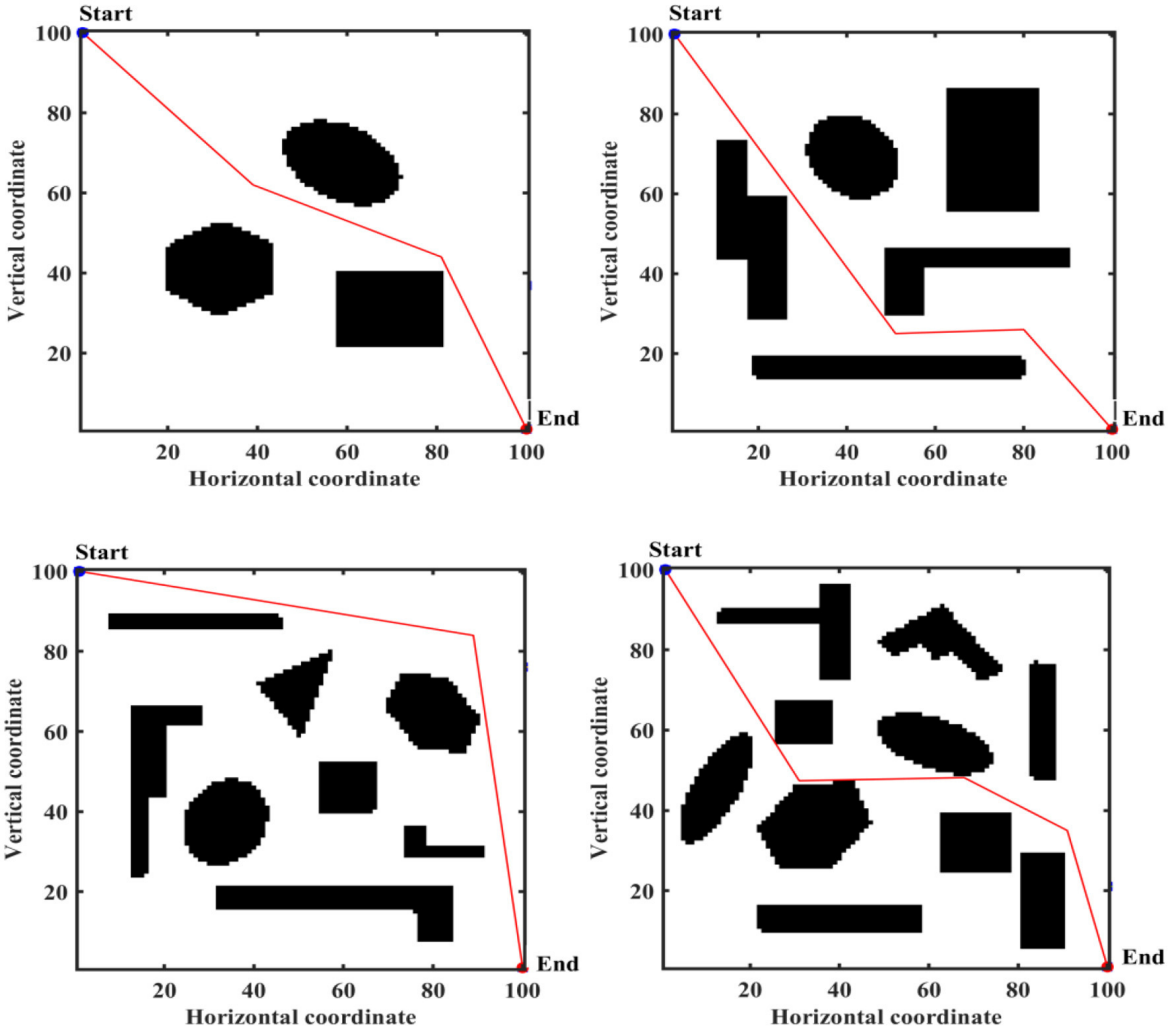
Path planning results of mobile robot in 4 different environments based on ABC algorithm for case 2.

From Table 4 and Figures 12–**16** we can find that the path length of the IMOABC algorithm-based path planning is the shortest in all four environments. That is, the path length performance of path planning based on the IMOABC algorithm is better than the path length performance of path planning based on the MOABC algorithm and ABC algorithm.

**Table 4:** The second experiment is to compare the path planning data between each algorithm.

**Performance indicators**	**Working environment**		**IMOABC**	**MOABC**	**ABC**
Path length	Map 1	Max	183.036	166.022	**150.4454**
		Min	**146.967**	155.866	150.4454
	Map 2	Max	200	166.033	**151.1716**
		Min	**142.005**	146.41	151.1716
	Map 3	Max	199.679	**149.641**	173.1685
		Min	**144.449**	146.547	173.1685
	Map 4	Max	193.988	161.705	**159.2363**
		Min	**150.584**	159.319	159.2363
Path security	Map 1	Max	**18.6376**	4.09792	0.9205
		Min	**1.11984**	0.974716	0.9205
	Map2	Max	**10.2965**	2.83367	0.575
		Min	0.00602009	0	**0.575**
	Map 3	Max	**8.46905**	1.04332	1.115
		Min	0	0.479438	**1.115**
	Map 4	Max	**3.32034**	0.890569	0.0625
		Min	0	**0.652126**	0.0625
Number of possible routes	Map 1		**39**	29	1
	Map 2		**25**	4-15	1
	Map 3		**32**	27	1
	Map 4		14	**30**	1

The meaning of the bold values is the optimal value.

From Table 4 and Figures 12–16 we can find that the security of IMOABC algorithm-based path planning is highest only in the map 1 environment for the shortest path length. That is, the path safety of path planning based on the IMOABC algorithm is better than the path safety of path planning based on the MOABC algorithm and ABC algorithm. In the map 2 environment, it is clear from Figure 13 that the safety performance of the IMOABC algorithm-based path planning is the most superior. In the map 3 environment, the security of path planning based on the IMOABC algorithm is not as good as the security of path planning based on the MOABC algorithm, but better than the security of path planning based on the ABC algorithm. In the map 4 environment, the best safety performance of path planning based on the IMOABC algorithm can be found in Figure 15. In summary, we can find that the path safety of IMOABC algorithm-based path planning is generally better than the security of MOABC and ABC algorithm-based path planning.

From Table 4 and Figures 12–16 we can find that the number of feasible routes for path planning based on the IMOABC algorithm is more than that based on the MOABC algorithm and ABC-based algorithm in map 1, map 2, and map 3 environments. And the IMOABC algorithm-based path planning feasible routes are a little more dispersed than the MOABC and ABC algorithm-based path planning feasible routes, which means that the IMOABC algorithm-based path planning has more options in choosing feasible routes according to its situation. In the map 4 environment, although the number of feasible routes of IMOABC algorithm-based path planning is inferior to that of MOABC algorithm-based path planning in the experiment, the feasible routes of MOABC algorithm-based path planning are very concentrated, which is inferior to the feasible routes of IMOABC algorithm-based path planning that can provide many choices, so IMOABC algorithm-based path planning for mobile robots is still the most superior in terms of the number of feasible routes.

In summary, we can find that the experimental results still demonstrate the superiority of the IMOABC algorithm-based mobile robot path planning and its robustness in terms of path length, path safety, and the number of feasible routes when the starting and ending points of the mobile robot are changed.

## 5. Conclusion

This thesis focuses on optimizing the path-planning problem of mobile robots based on the IMOABC algorithm. Mobile robot path planning is a complex optimization problem. First of all, it is necessary to ensure that the mobile robot does not collide with obstacles in the working environment map, but also to ensure that the mobile robot can go from the starting point to the endpoint smoothly and that the performance indicators of the path planning should be as good as possible. In this thesis, an IMOABC algorithm is proposed in conjunction with the MOABC algorithm for solving mobile robot path planning problems. Firstly, the external archive pruning strategy, non-dominated sorting and crowding distance strategy, search strategy, and Pareto optimal strategy are introduced into the MOABC algorithm, which leads to the IMOABC algorithm. The IMOABC algorithm is also compared with three other similar algorithms for verification, and the results show the effectiveness and stability of the IMOABC algorithm. The IMOABC algorithm is then applied to mobile robot path planning based on two performance optimization metrics selected for path planning, and the results show that the algorithm can complete the given path planning task. And compare the experiments with two other algorithms (MOABC algorithm and ABC algorithm) on the Matlab platform to verify the effectiveness and efficiency of the IMOABC algorithm in mobile robot path planning. Our current limitation is simulation validation, and future work will be conducted in physical experiments.

## Data availability statement

The original contributions presented in the study are included in the article/supplementary material, further inquiries can be directed to the corresponding author.

## Author contributions

QC completed the improvement of the algorithm and analysis. QC and HD wrote the first draft. XM proposed the idea and directed the experiment. PL assisted in the debugging of the algorithm. HW provided facilities and equipment. XM helped the complete the revised manuscript. All authors contributed to the article and approved the submitted version.
